# The impact of the expansion of urban vegetable farming on malaria transmission in major cities of Benin

**DOI:** 10.1186/1756-3305-3-118

**Published:** 2010-12-12

**Authors:** Anges Yadouléton, Raphael N'Guessan, Hyacinthe Allagbé, Alex Asidi, Michel Boko, Razack Osse, Gil Padonou, Gazard Kindé, Martin Akogbéto

**Affiliations:** 1 Centre de Recherche Entomologique de Cotonou (CREC), 06 BP 2604 Cotonou, République du Bénin, Tél. (+229) 21330825; 2 London School of Hygiene and Tropical Medicine, Kepel Street, WC1E 7HT, London, UK; 3 Université d'Abomey-calavi, Department of Geography, Cotonou, République du Bénin; 4 Faculté des Sciences de la Santé, Université Nationale du Bénin, République du Benin

## Abstract

**Background:**

Urban agricultural practices are expanding in several cities of the Republic of Benin. This study aims to assess the impact of such practices on transmission of the malaria parasite in major cities of Benin.

**Method:**

A cross sectional entomological study was carried out from January to December 2009 in two vegetable farming sites in southern Benin (Houeyiho and Acron) and one in the northern area (Azèrèkè). The study was based on sampling of mosquitoes by Human Landing Catches (HLC) in households close to the vegetable farms and in others located far from the farms.

**Results:**

During the year of study, 71,678 female mosquitoes were caught by HLC of which 25% (17,920/71,678) were *Anopheles *species. In the areas surveyed, the main malaria parasite, *Plasmodium falciparum *was transmitted in the south by *Anopheles gambiae *s.s. Transmission was high during the two rainy seasons (April to July and October to November) but declined in the two dry seasons (December to March and August to September). In the north, transmission occurred from June to October during the rainy season and was vehicled by two members of the *An. gambiae *complex: *Anopheles gambiae s.s*. (98%) and *Anopheles arabiensis *(2%).

At Houeyiho, Acron and Azèrèkè, the Entomological Inoculation Rates (EIRs) and the Human Biting Rates (HBRs) were significantly higher during the dry season in Households Close to Vegetable Farms (HCVF) than in those located far from the vegetable areas (HFVF) (p < 0.05.). However, there were no significant differences in HBRs or EIRs between HCFV and HFVF during the rainy seasons at these sites (p > 0.05).

The knock-down resistance (*kdr*) mutation was the main resistance mechanism detected at high frequency (0.86 to 0.91) in *An. gambiae *s.l. at all sites. The *ace-1^R ^*mutation was also found but at a very low frequency (< 0.1).

**Conclusion:**

These findings showed that communities living close to vegetable farms are permanently exposed to malaria throughout the year, whereas the risk in those living far from such agricultural practices is limited and only critical during the rainy seasons. Measures must be taken by African governments to create awareness among farmers and ultimately decentralize farming activities from urban to rural areas where human-vector contact is limited.

## Introduction

The geographical distribution of malaria so far described in sub-Saharan Africa is diverse. This ranged from savannah malaria, forest, highland, urban and hydro-agricultural malaria [1]. Currently, the need to investigate urban malaria has become urgent due to the resurgence of the disease and the agro-economical interest by populations in developing subsistence activities in urban and suburban areas of major cities in sub-Saharan Africa [2-6]. In recent studies by Robert *et al*. [3] and Warren *et al*. [7] it was shown that urbanization decreases malaria prevalence as a results of a drastic reduction in *Anopheles *breeding sites better access to treatment and improved (mosquito-proof) housing measures (overview in [8]). It is therefore important to emphasize the role played by urbanization in reducing malaria transmission by twofold [9]. Furthermore, agricultural activities play an important role in malaria transmission in both urban and peri-urban zones [6]. Indeed, poverty, food insecurity and malnutrition have become urban issues in sub-Saharan Africa. While meeting these challenges in cities of sub-Saharan Africa is critical, it represents a serious issue of public health [10]. Agro-economic practices involving vegetable farming is now common in many urban areas and this provides suitable breeding sites for mosquitoes with potentially higher epidemiological risk of malaria in urban than rural areas.

The general trend in practice now is that, usually non-used spaces (marshland, road edges, beaches etc) are increasingly transformed into vegetable farms comprising different kinds of crops. In Cotonou the capital city of Benin, peri-urban agriculture consists of belts of vegetable farming surrounding the city.

The advantages of urban agriculture are considerable. They contribute to improve the living conditions of citizen by supplying food, income and employment [11].

However, the economic and social value of urban and peri-urban agriculture is hindered by a number of factors including the proliferation of mosquito breeding sites. Many studies have reported the relationship between malaria and rice production, but little is known about the link between malaria transmission and urban agriculture. A recent study conducted by Robert *et al*. [8] has shown that vegetable farming in urban areas of Dakar, (Senegal) might not be the suitable breeding sites for larval development.

However, Matthys *et al*. [12] found that peri-urban agriculture created more breeding sites for *Anopheles *and therefore increased malaria risk in the city. An entomological study conducted in Ghana showed higher *Anopheles *biting rates in urban areas with agriculture compared to urban areas without such practice [13]. Another study in Kenya found no association between urban farming at household level and vector breeding sites [14].

In Benin, there are few data available that investigate the association between malaria transmission and urban vegetable farming.

The present study was conducted in three major sites of urban farming in Benin with the aim of investigating the entomological aspects of malaria transmission in relation to seasonal variations of vector populations in these areas of Benin.

Specifically, the study aimed to determine (a) the distribution of *Anopheles *mosquito species throughout the year at these sites, (b) their human biting pattern, (c) the infectivity rates of malaria vectors and (d) the entomological inoculation rates and malaria transmission in the three study areas.

## Methods

### Study area

**Figure 1:**
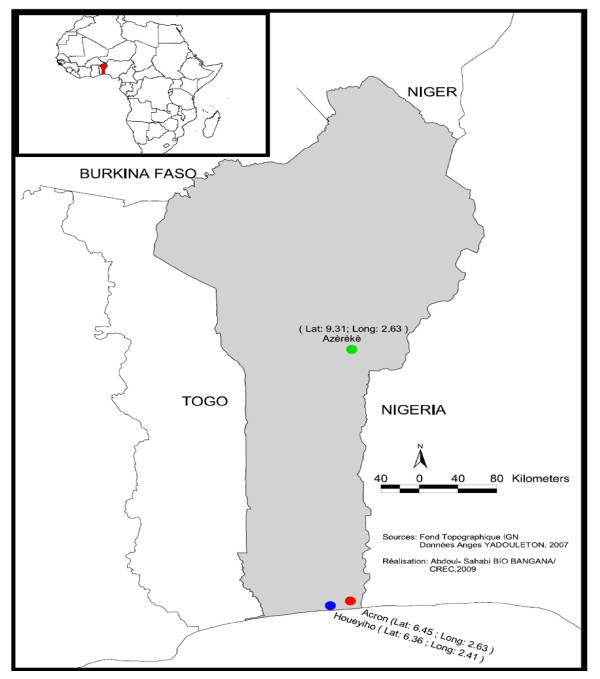
**Map of Benin showing the three study sites**.

The study was conducted in Benin, from January 2009 to December 2009 in three vegetable farms (Figure 1):

(i) Houeyiho in Cotonou, (economic capital city of Benin). The vegetable farm is located at 6°45'N and 2°31'E in a highly populated zone. The farm is 14-hectares in size and shared between five local cooperatives of approximately 2,000 farmers.

(ii) Acron in Porto-Novo, (administrative capital city of Benin)

The vegetable farm is at 6°30'N and 2°47'E, at the outskirt of Porto-Novo and has the longest history of vegetable farming in the region. Initially it consisted of three hectares but has recently expanded up to 20 hectares. The number of farmers has also increased to about 150 individuals.

(iii) Azèrèkè, Parakou. This farm is located at 9°22'N and 2°40'E at the vicinity of Parakou city, known as the Azèrèkè site. The size of this vegetable plantation is 10 hectares.

Agricultural practices in those farms create numerous trenches that retain rain and water from irrigation systems. These stagnant waters provide suitable breeding sites for mosquitoes, particularly *Anopheles gambiae*, the main malaria vector in the areas.

### Field mosquito collection

Indoor collections of adult mosquitoes were carried out monthly from January to December 2009. Collections were organized in Households Close to Vegetable Farms (HCVF) and in others Far from the Farms (HFVF) where there is no agricultural practice.

Adult mosquitoes were collected using two sampling methods:

(1) Indoor and outdoor Human Landing Catches (HLC) performed monthly over two consecutive nights (8:00 PM to 6:00 AM), in 4 randomly selected compounds;

(2) Indoor Pyrethrum Spray Catches (PSC) in 4 other selected compounds; the same compounds in each sampling method being consistently used throughout the study. Collectors gave prior informed consent and received anti-malaria prophylaxis and yellow fever immunization. They were organized in teams of two for each collection point and they rotated between location within houses every two hours. Mosquitoes from HLC were used to evaluate the sporozoite infection rate of each molecular form. Knocked down mosquitoes falling on white bed sheets were preserved for identification at molecular level using PCR analysis for their resistance status as described by Martinez-Torres et *al*. [15]. The PSC were carried out monthly from December to January and used to establish the temporal dynamics of mosquito density and the molecular forms of *An. gambiae*.

All mosquitoes were kept separately in labelled tubes containing silica gel and frozen at -20°C for further laboratory analysis.

#### Laboratory processing of mosquitoes

Based on morphological characters using standard identification keys [16], all female mosquitoes referring to *An. gambiae *complex were identified. The head-thoraces of these females from the human landing catches were tested for the presence of CircumSporozoite Protein (CSP) of *P. falciparum *using enzyme-linked immunosorbent assay (ELISA) as per Wirtz *et al*. [17]. Identification of species and characterization of molecular forms within the *An. gambiae *complex were performed using PCR-RFLP [18].

#### Entomological parameters

The entomological indicators of malaria parasite transmission intensity at the sites were:

(1) the human biting rate (HBR), which is the number of mosquitoes biting a person during a given time period (bites/p/t) (time being night, month or year);

(2) the CSP rate is the proportion of mosquitoes found with *Plasmodium falciparum *CSP over the total number of mosquitoes tested:

(3) the Entomological Inoculation Rate (EIR), expressed as the number of infective bites of anopheline per person per unit of time (bi/p/t) and calculated as the product of the HBR by the CSP rate.

### Data analysis

An analysis of variance (ANOVA) was performed to compare the entomological estimates (HBRs, EIRs) between the seasons at the sites in the North and south Benin.

The resistance allele frequency at the *kdr *and *Ace-*1 locus was calculated using Genepop software (version 3.3) as described by Raymond and Rousset [19].

#### Ethical considerations

Ethical approval for this study was granted by the Ethical Committee of the Ministry of Health in Benin. Verbal consent was asked to the head of each household for the spray catches and consent of collectors was obtained prior to HLC. In case of refusal, permission was sought from the next household.

## Results

### Mosquito fauna composition

A total number of 71,678 mosquitoes were collected from HLC and 29,295 from PSC at the three study sites (Table 1). The majority was *Culex spp *(74%). Of the remaining 26%, 97% were *Anopheles gambiae *s.l. and 3% were *An. pharoensis, Anopheles ziemanni*, and *An. funestus *all together.

**Table 1:** Mosquitoes collected by human landing catches (HLC) and pyrethrum spray catches (PSC) at the study sites.

	Houeyiho		Acron		Azéréké	
Species	HLC	PSC	HLC	PSC	HLC	PSC
Total mosquitoes caught	28,654	9,879	25,852	10,495	17,172	8,921
Total *Culex *Spp	20,7	8,6	19	9,1	13,2	7,4
Total *Anopheles *Spp	7,8	1,3	6,7	1,2	3,8	1,4
*An. gambiae s.l*	7,6	1,2	6,5	1,1	3,8	1,2
*An. pharoensis*	60	23	38	18	12	29
*An. ziemanni*	158	45	112	76	45	28
*An. funestus*	2	0	5	1	0	0
Total *Aedes *spp	130	34	138	61	94	78
Total *Mansonia *spp	23	20	54	56	15	22

#### Dynamic of the molecular forms

PCR species from *An. gambiae *s.l. collected revealed the presence of two members of *An. gambiae s.l*. at Azèrèkè: *An. gambiae *s.s., *and An. arabiensis *(Table 2). At Houeyiho and Acron, the entire mosquito population was *An. gambiae *s.s of the M molecular form throughout the year. However, at Azèrèkè, *An. gambiae s.s*. predominates at 85%. Despite the presence of both *An. gambiae *and *An. arabiensis *identified at Azèrèkè, the PCR analysis revealed two molecular forms in *An. gambiae *s.s. only, with proportions of 65% for the M and 35% for the S form.

**Table 2:** Summary results of PCR analysis of An. gambiae s.l. collected at the study sites.

	PCR form			PCR Species		
	**Total tested**	**%S**	**%M**	***% An. gambiae***	***% An. melas***	***% An. arabiensis***

Houeyiho	360	-	100	100	-	-
Acron	360	-	100	100	-	-
Azèrèkè	360	65	35	85	-	15

- = Not found

### Seasonal abundance and biting rates

**Figure 2:**
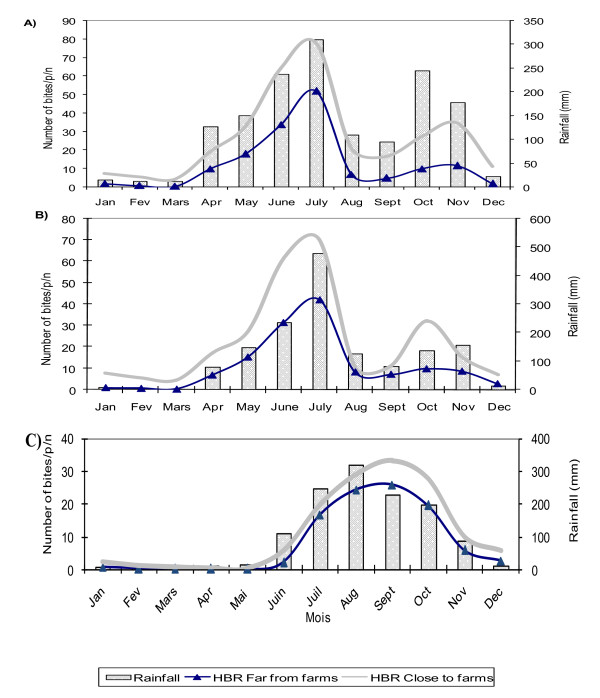
**Number of Human bites/person/night and rainfall at Houeyiho (A), Acron (B) and Azèrèkè (C) from January to December 2009**.

The Annual Human Biting Rate (HBR) was estimated from the Human Landing Catches (HLC). The highest bites of *An. gambiae s.l*. during the rainy seasons was found in July (80 bites/p/n) in southern Benin and September (100 bites/p/n) in the northern part of the country as well (Figure 2).

The results from this study showed that the average HBR of *An. gambiae s.l*. was 10.95 bites/p/n at Houeyiho; 7.84 bites/p/n at Acron and 3.04 bites/p/n at Azèrèkè during the dry season in HCVF. These bites from HCVF were significantly higher than in HFVF at Houeyiho (2.70 bites/p/n), Acron (2.85 bites/p/n) and Azèrèkè (1.41 bites/p/n) (P < 0.05).

The human population living in HCVF received about one to five times higher bites of *An. gambiae s.l*. than those who lived in HFVF. However, there was no significant difference between the HBRs during the rainy season in HCFV and HFVF at Houeyiho, Acron and Azèrèkè (P > 0.05 for the three sites). The average annual HBR from January through December was significantly higher in HCVF than that from HFVF at the three sites (P < 0.05) (Table 3).

**Table 3:** Mean number of An. gambiae s.l. bites per person per night (Mean bi/p/n) during the seasons and annually, as determined by human landing Catches (HLC) at the study sites

	Houeyiho			Acron			Azèrèkè		
	**Dry season**	**Rainy season**	**annual**	**Dry season**	**Rainy season**	**annual**	**Dry season**	**Rainy season**	**annual**

Mean bites/p/n*	10.95	42.73	9,798	7.84	37.34	8,224	3.04	23.14	6,1671
Mean bites/p/n**	2.70	22.42	4,563	2.85	19.01	4,601	1.41	17.75	3,000
***P *value**	0.015	0.115	0.091	0.033	0.137	0.024	0.033	0.428	0.025

* = close to farms

** = far from farms

#### Sporozoite rate and EIR

A low percentage of *Anopheles *caught from the HLC (1.6%) was circumsporozoite protein positive at the three study sites. The main malaria parasite was *P. falciparum *transmitted by *Anopheles gambiae *s.l. in the southern sites surveyed. Transmission at these sites was high during the two rainy seasons (April to-July and October to November) and low during the two dry seasons (December to March and August to September) (Figure 2). In the north of the country, transmission occurred during the rainy season (June to October), with at least two members of *An. gambiae s.l*.: *An. arabiensis *(15%) and *An. gambiae s.s*. (85%) transmitting *P. falciparum*.

The EIRs were significantly higher in the dry season in HCVF at Houeyiho (0.28 bi/p/n), Acron (0.23 bi/p/n) and at Azèrèkè (0.15 bi/p/n) than in HFVF at the same sites Houeyiho (0.041 bi/p/n); Acron (0.01 bi/p/n) and Azèrèkè (0.031 bi/p/n) (P < 0.05).

The trend in average annual EIRs at the three sites was similar to that observed in the dry season, with significantly higher EIR in HCVF than in HFVF at the three sites (P < 0.05) (Table 4). However, during the rainy season, there was no significant difference between the EIRs from HCFV and HFVF (P > 0.05 for the three sites).

**Table 4:** Entomological Inoculation rates (EIR) recorded at the study sites

	Houeyiho			Acron			Azèrèkè		
	**Dry season**	**Rainy season**	**annual**	**Dry season**	**Rainy season**	**annual**	**Dry season**	**Rainy season**	**annual**

EIR (bi/p/n)*	0.28	0.33	169.18	0.23	0.38	112.16	0.15	0.36	95.52
EIR (bi/p/n)**	0.041	0,194	64.64	0.01	0.25	65.39	0.031	0.19	39.92
***P *value**	0.000	0.093	0.038	0.033	0.137	0.024	0.033	0.428	0.025

* = number of infective bites per person per night in households close to farms

** = number of infective bites per person per night in households far from farms

#### Distribution of the *kdr *and *ace-1R *mutations

An average of 30 mosquitoes from indoor resting fauna were analysed every month for the Leu-Phe *kdr *and *ace-1^R ^*mutations (Table 5). In southern Benin, the *kdr *mutation was found in all M form of *An. gambiae*, with a frequency of 0.89 at Acron and 0.91 at Houeyiho.

**Table 5:** PCR analysis of the kdr and ace-1 allelic frequency in An. gambiae s.l. collected at the study sites

		*kdr*				*ace-1*			
	**Total tested**	**RR**	**RS**	**SS**	**frequency**	**RR**	**RS**	**SS**	**frequency**

Houeyiho	360	305	48	7	0.91	0	2	0	0.002
Acron	360	298	45	17	0.89	0	1	0	0.001
Azèrèkè	360	285	53	22	0.86	0	3	0	0.004

At Azèrèkè, in the north, *Kdr *occurred both in the M and S forms but at a higher frequency in S (75%) than M form (25%) (*P *< 0.05).

The *ace-1^R ^*mutation was also found at all sites surveyed but at a very low frequency. It was 0.02 at Houeyiho, 0.01 at Acron and 0.04 at Azèrèkè (Table 5).

## Discussion

The findings from the present study showed a clear evidence of the dynamics of malaria transmission in urban or sub-urban areas of Benin where vegetable farming activities have grown extensively.

The abundance and fluctuations in larval and adult densities of samples collected were inherent to the environmental and ecological characteristics related to each site assessed [20].

Indeed, the diversity of anopheline species found in this study either in the south or northern part of Benin showed that apart from *Anopheles gambiae *s.s., the major vector of malaria parasites in West Africa, *An. arabiensis *should also be considered as a secondary transmitter in northern Benin though at a lesser extent. Yadouleton et *al*. [10] reported the presence of *An. arabiensis *in the Northern part of Benin at low frequency. Furthermore, within the *An. gambiae *complex, the S form was only found at the Azèrèkè site in the North (Sudano-guinean ecotype) while the M form was identified at Houeyiho and Acron in the coastal area of Cotonou (guinean ecotype) and the northern sub-urban areas. Corbel et *al*. [21] in Benin and Awolola *et al*. [22] in Nigeria reported that the geographical distribution correlated with the ecological or climatic factors as the M form is more adapted to the dry environment and breeds along irrigated fields than the S form which is commonly found in humid forest areas and temporary pools. The predominance of *An. gambiae *s.l. in the study area is consistent with its distribution throughout Africa.

The presence of higher biting rates (Figure 2) and sporozoite infective *Anopheles *(Table 4) in households close to vegetable farms than in those on the far side of the farms have shown that malaria parasite transmission was permanent during the year and was reinforced by the presence of breeding sites in urban vegetable farming. In fact, results from this study showed that in the three study sites, people who lived nearby the vegetable farms received during the dry season one to five times more bites than those who lived farther, but during the rainy season, there was no significant difference between HCVF and HFVF. The increased number of biting (HBR) and EIR recorded during the dry seasons in houses close to the vegetable farms compared to those far from the farms could be explained by the presence of permanent pools and puddles maintained during watering of vegetable crops.

These findings showed that communities living close to vegetable farms are permanently exposed to malaria throughout the year whereas the risk in those living far from such agricultural practices is limited and only critical during the rainy seasons.

The main mechanism conferring resistance in *An. gambiae *to pyrethroids (*Leu-Phe kdr *mutation) in West Africa was found in mosquito samples collected in different sites.

The allelic frequency of this mutation among populations collected near or far from the vegetable farms was high (> 0.90). This could be the direct consequence of the extensive use of pesticides for cotton crop protection in the south and northern Benin [23,24] or the use of the same pesticides by local farmers against vegetable pests [10]. The gene frequency being already too high in both situations, it appeared difficult to separate the effect of the local farming practices from that of the old history of cotton production and pesticides on resistance selection in the areas surveyed. Distinguishing the impact of the two agricultural practices will require sampling and testing of mosquitoes in areas where *kdr *is still on its moderate form in Western Africa.

## Competing interests

The authors declare that they have no competing interests.

## Authors' contributions

AY carried out field experiments, collected, analysed, interpreted data and wrote the manuscript. NR and AA contributed to the design of the study and revised the manuscript for intellectual content, BM, KD contributed to the design of the study. GP and RO and HA helped with the field activities. MA conceived, designed the study, and revised the manuscript for intellectual content.

All authors read and approved the final manuscript.
